# Plastome structure and phylogeny of *Gnetum luofuense* C.Y. Cheng (Gnetaceae, Gnetales)

**DOI:** 10.1080/23802359.2020.1821818

**Published:** 2020-09-18

**Authors:** Aimee Caye G. Chang, Mary Ann C. Bautista, Yan Zheng, Tao Wan, Tao Chen

**Affiliations:** a South China Botanical Garden, Chinese Academy of Sciences, Guangzhou, P. R. China; b Shenzhen Fairy Lake Botanical Garden, Chinese Academy of Sciences, Shenzhen, P. R. China; c Graduate School, University of Chinese Academy of Sciences, Beijing, P. R. China

**Keywords:** Gnetaceae, Gnetales, *Gnetum luofuense*, complete chloroplast genome, plastome

## Abstract

This article describes the complete chloroplast genome of *Gnetum luofuense*. The *G. luofense* plastome was 114,795 bp in length, containing a large single copy region (66,103 bp) and a small single copy region (9438 bp), separated by two inverted repeat regions (19,627 bp). The genome lost all *ndh* genes and contained 116 genes, including 68 protein-coding genes, 40 *tRNA* genes, and eight *rRNA* genes. The GC content was 33.3%, 12 genes all contained an intron, *ycf3* gene contained two introns while *rps12* was a transpliced gene. Phylogenetic analysis using 61 concatenated protein-coding genes suggests that *G. luofuense* with the rest of other gnetophytes were sister to or nested within all conifers.

Gnetales is a small group of gymnosperms comprising three genera: *Gnetum*, *Ephedra*, and *Welwitschia*. Among extant seed plants, the phylogenetic placement of Gnetales remains controversial (Ran et al. 2018). Thus, various hypotheses regarding its exact taxonomic position were proposed. This includes the ‘gnepine’ (sister to pines), ‘gnetifer’ (sister to all conifers), ‘gnecup’ (sister to non-Pinaceae cupressophytes), and ‘anthophyte’ (sister to angiosperms) hypotheses (Zhong et al. 2010). *Gnetum luofuense* C.Y. Cheng is one of the lianoid species of *Gnetum* distributed in the South Eastern (SE) part of China. Morphologically, it is differentiated from other species of the genus by the presence of 9–11 involucral collars in male spikes and coarsely reticulate-wrinkled seeds (dried) (Fu et al. 1999). Currently, it is classified as Near Threatened (NT) by the IUCN Red List of Threatened Species (Baloch 2011; IUCN 2020). Chloroplast genomes, herewith referred to as plastomes, are often used in phylogenetic research because they are highly conserved and less complex compared to nuclear and mitochondrial DNA but still hold sufficient genomic data despite having relatively smaller dimensions (Chang et al. 2020). At present, six completely sequenced chloroplast genomes under the genus *Gnetum* were deposited in NCBI GenBank and the addition of *G. luofuense* aimed to contribute additional sequence data for future studies resolving issues in Gnetales phylogeny and seed plants evolution.

Fresh leaves of *G. luofuense* were collected from Sanzhaigu, Yonghan, Longmen County, Guangdong, China (Chen et al. 2018123101, SZG, N29°35′30.9′′, E113°56′2.1′′). DNA was extracted using SDS method (Shi et al. 2012) and stored at −80 °C prior to sequencing using Illumina HiSeq 2500 platform. Paired-end sequences with an average read length of 150 bp were generated with an average sequencing depth of 2164.6X. After filtering reads, the final circular plastome was de novo assembled using SPAdes version 3.11.0 (Vasilinetc et al. 2015) and was annotated using CpGAVAS (Liu et al. 2012), RNAmmer version 1.2 Server (Lagesen et al. 2007), and tRNAscan-SE (Chan and Lowe 2019) for protein-coding genes, rRNA and tRNA annotations, respectively. The complete nucleotide sequence of *G. luofuense* plastome was deposited in NCBI GenBank with accession number MT663149.

The plastome of *G. luofuense* has a circular quadripartite structure with a total genome length of 114,795 bp. It contained a large single copy region (66,103 bp) and a small single copy region (9438 bp), separated by two inverted repeat regions (19,627 bp). The genome lost all *ndh* genes and contained 116 genes, including 68 protein-coding genes, 40 *tRNA* genes, and eight *rRNA* genes. The GC content of *G. luofuense* plastome is 33.3%. The genes *trnK-UUU*, *trnG-UCC*, *atpF*, *rpoC1*, *trnL-UAA*, *trnV-UAC*, *petB*, *petD*, *rpl16*, *rpl2*, *trnI-GAU*, and *trnA-UGC* all contained one intron, the *ycf3* gene contained two introns while *rps12* gene was found to be transpliced.

To determine the phylogenetic position of *G. luofuense* within seed plants, the complete plastome sequence was aligned with other 19 species representing basal angiosperms and gymnosperms (Cycadales, Coniferales, and Ginkgoales). For Gnetales, at least two species per genus were selected for phylogenetic analysis with the exception of the monotypic *Welwitschia*. Maximum-likelihood tree (Figure 1) was constructed using GTR + Gamma substitution model and 1000 bootstrap replicates using built-in RAxML in CIPRES (Miller et al. 2010). Using *Psilotum nudum* (Psilotales) as outgroup, phylogenetic tree indicated that *G. luofuense* and *Gnetum parvifolium* were closest to the genus *Welwitschia* diverging and forming a distinct clade with *Ephedra*. Moreover, all three Gnetales genera were phylogenetically placed within all conifers, similar to the study conducted by Wan et al. (2018). Hence, our chloroplast genome topology was closest to the ‘gnetifer’ hypothesis but not congruent with ‘gnepine,’ ‘gnecup,’ or ‘anthophyte’ hypotheses.

**Figure 1.**
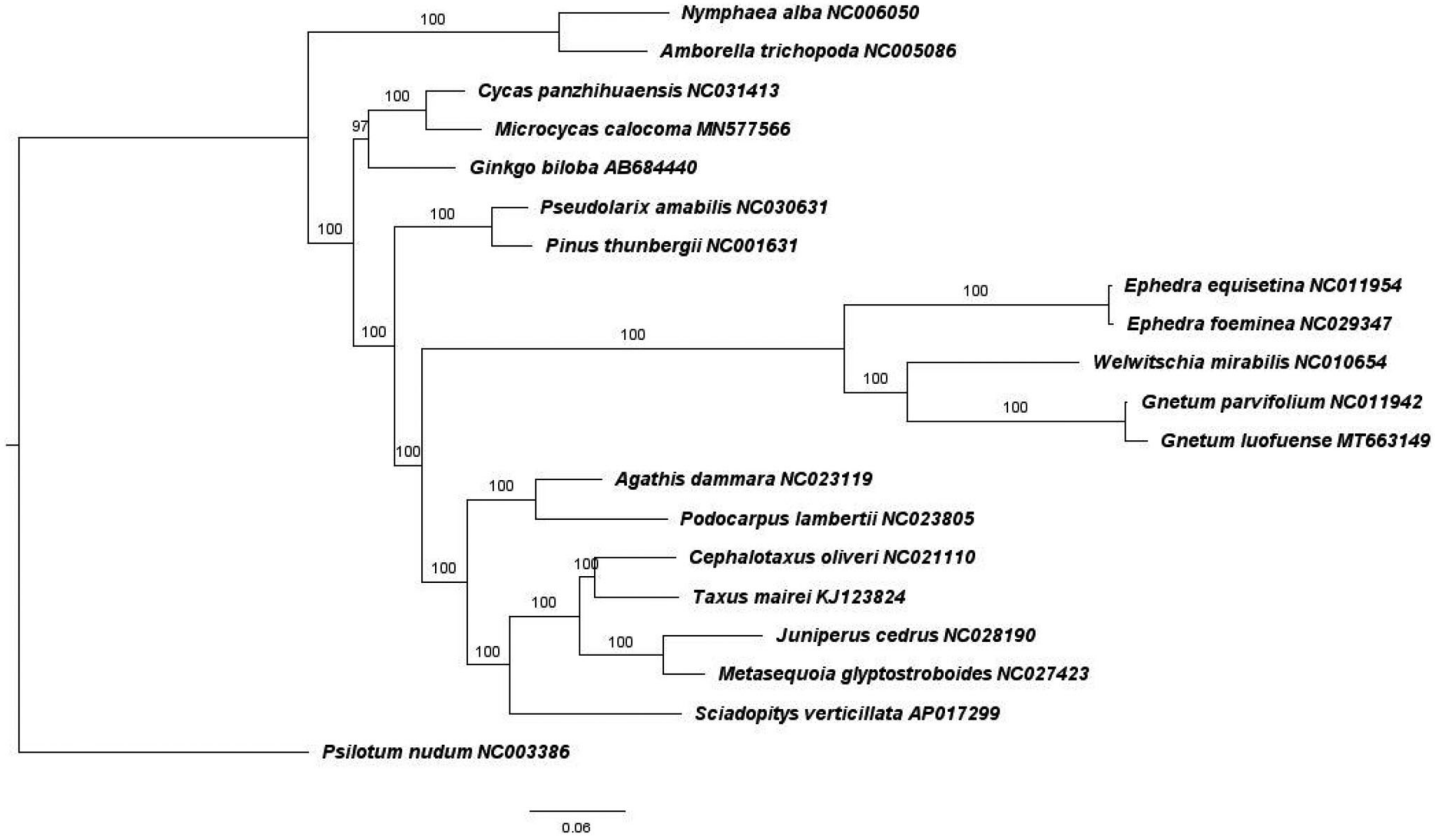
Maximum-likelihood tree showing phylogenetic placement of *G. luofuense* within extant seed plants.

## Data Availability

All chloroplast genome sequences generated and used in this study are accessible via NCBI GenBank database. The complete nucleotide sequence of *G. luofuense* plastome was deposited in NCBI GenBank with accession number MT663149 (https://www.ncbi.nlm.nih.gov/nuccore/MT663149).
